# Association Between Nap Duration and Cognitive Functions Among Saudi Older Adults

**DOI:** 10.3389/fnins.2022.917987

**Published:** 2022-06-03

**Authors:** Yousef D. Alqurashi, Khalid AlHarkan, Adam Aldhawyan, Ahmed Bahamdan, Assim Alabdulkader, Raed Alotaibi, Saleh Alduailej, Mohammed Alqahtani, Kholoud Al Ghamdi

**Affiliations:** ^1^Respiratory Care Department, College of Applied Medical Sciences, Imam Abdulrahman Bin Faisal University, Dammam, Saudi Arabia; ^2^Department of Family and Community Medicine, College of Medicine, Imam Abdulrahman Bin Faisal University, Dammam, Saudi Arabia; ^3^Department of Physiology, College of Medicine, Imam Abdulrahman Bin Faisal University, Dammam, Saudi Arabia

**Keywords:** nap, cognitive function, elderly, sleep, geriatrics

## Abstract

**Purpose:**

Current evidence of whether napping promotes or declines cognitive functions among older adults is contradictory. The aim of this study was to determine the association between nap duration and cognitive functions among Saudi older adults.

**Methods:**

Old adults (> 60 years) were identified from the Covid-19 vaccine center at Imam Abdulrahman Bin Faisal University, Dammam, Saudi Arabia between May and August 2021. Face-to-face interviews were conducted by a geriatrician or family physicians. Data collected for each participant included sociodemographic, sleep patterns, health status and cognitive functions. St. Louis University mental status (SLUMS) was used to assess the cognitive functions. A multi-Linear regression model was used to determine the association between cognitive functions and nap duration.

**Results:**

Two-hundred participants (58 females) aged 66 ± 5 years were recruited. Participants were categorized according to their nap duration into non-nappers (0 min), short nappers (> 0- ≤ 30 min), moderate nappers (> 30–≤ 90 min), and extended nappers (> 90 min). The mean duration of the nap was 49.1 ± 58.4 min. The mean SLUMS score was 24.1 ± 4.7 units. Using the multi-linear regression model, the mean total SLUMS score for extended nappers was, on average, significantly lower than non-nappers [−2.16 units; 95% CI (−3.66, −0.66), *p* = < 0.01] after controlling for the covariates (age, sex, education level, sleep hours, diabetes mellitus, hypertension, pain).

**Conclusions:**

Extended napping was associated with deterioration in cognitive function among Saudi older adults.

## Introduction

Advanced age is associated with many physiological changes in sleep architecture, one of which is increased daytime nap. It is considered one of the healthy lifestyle practices and very common in some cultures and in the elderly compared to other age groups (Bahammam et al., 2005; Leng et al., 2019; Al-Abri et al., 2020). Although current evidence showed association between daytime nap duration and cardiovascular diseases, diabetes, and overall mortality (Zhong et al., 2015; Leng et al., 2016), the evidence on the relationship between daytime nap duration and cognitive function among older adults is still scarce (Leng et al., 2019).

A number of studies investigated the association between daytime nap duration and cognition in older adults (Lovato and Lack, 2010; Zhang et al., 2020). Kitamura et al. (2021) found that short daytime naps (< 30 min) were associated with reduced risk of cognitive decline among community dwelling older adults (Kitamura et al., 2021). Another study by Li et al. (2017) found that afternoon nap of less than 90 min was beneficial for episodic memory, visuospatial abilities, and orientation to time and attention in Chinese older adults (Li et al., 2018). However, such benefit was not evident for motor tasks in a sample of German older adults (Backhaus et al., 2016). Furthermore, Scullin et al. (2017) examined the impact of 90-min daytime naps on cognition among younger versus older adults. They showed that younger adults benefited from daytime naps, and such benefits diminished with advancing age (Scullin, 2013). A recent literature review showed that the current evidence is not enough to determine the exact role of napping in the health of older adults (Zhang et al., 2020).

The aim of this study was to determine the association between nap duration and cognitive function among Saudi old adults. We hypothesized that there will be an association between cognitive decline and nap duration in such a population.

## Materials and Methods

### Participants

This cross-sectional study was conducted between May and August 2021.

Participants were recruited from the vaccine center at Imam Abdulrahman Bin Faisal University, Dammam, Saudi Arabia. Inclusion criteria were individuals 60 years or older. Participants were excluded from the study if they reported recent head trauma in the past 3 months. All participants gave written informed consent, and the study was approved by the Institutional Review Board of Imam Abdulrahman bin Faisal university (IAU) (IRB Number -2021-01-129).

### Measurements

Questionnaires were completed using face-to-face interviews by either a geriatrician or a family medicine trainee. The family medicine trainees were trained by the geriatricians in how to conduct the interview. The questionnaire collected data on the participants’ sociodemographic, sleep pattern including total sleep time at night and health status including previous diagnosis with diabetes mellitus, hypertension, coronary artery diseases. We included information about stroke, depression, and anxiety, but we did not include them in the analysis due to the fact that a very few numbers of individuals had these conditions. A validated Arabic version of Athens insomnia scale, STOP-BANG questionnaire and St. Louis University mental status (SLUMS) examination were used (Szcześniak and Rymaszewska, 2016).

#### Athens Insomnia Scale

This scale is an 8-item questionnaire that measures the intensity of sleep difficulties. The eight questions pertain to sleep induction, early awakening and awakening during the night, total sleep duration, sleep quality and daytime wellbeing, functioning capacity and sleepiness. A cut-off score of ≥ 6 points was used to detect insomnia.

#### STOP-BANG

A questionnaire consists of eight dichotomous (yes/no) items related to clinical features of obstructive sleep apnea (OSA). The total score ranges from 0 to 8. Patients with a STOP-BANG score of 0 to 2 can be classified as low risk for presence of OSA whereas those with a score of 3 to 8 can be classified as high risk for presence of OSA.

#### The St. Louis University Mental Status Examination

The SLUMS is a 30-point, 11-item scale that is used to identify people with dementia or mild neurocognitive impairment. The total possible score is 30. Interpretation of the score depends on the level of education; High school education: Normal: 27–30; Mild neurocognitive disorder: 21–26; Dementia: 1–20. Less than high school education: Normal: 25–30; Mild neurocognitive disorder: 20–24; Dementia: 1–19. We used the validated Arabic version of the SLUMS in our study (Abdelrahman and El Gaafary, 2014).

### Statistical Analysis

Population characteristics were summarized across levels of daily napping durations; non-nappers (0 min), short nappers (0–≤30 min), moderate nappers (> 30–≤90 min), and extended nappers (>90 min). Mean and standard deviation were used for continuous variables, and count and percentage were used for categorical variables. ANOVA was used to test for differences across napping duration for normally distributed continuous variables, Kruskal–Wallis test for skewed variables, and chi-square test for categorical variables.

We used multivariable linear regression to study the association between napping duration and risk of developing cognitive impairment adjusting for known confounders including age, sex, education level, sleep hours, diabetes mellitus (DM), hypertension (HTN), pain, risk of insomnia and risk of sleep apnea. All statistical tests were two-sided, using *p*-value of 0.05 for statistical significance. Analyses were performed using (R version 4.1.1).

## Results

Two hundred individuals with a mean age of 66 ± 5.5 years participated in the study, of which 71% were males. Table 1 summarizes the population characteristics across napping duration categories. Of the participants, the majority were non-nappers (*n* = 75), followed by moderate nappers (*n* = 61). The mean SLUMS score for the whole population was 24.1 ± 4.7 units. Table one shows the participants’ demographic and cognitive scores.

**TABLE 1:** Demographic and cognitive scores among the participants.

	Non- nappers	Short- nappers	Moderate- nappers	Extended- nappers	Overall	*P*-value
	(*N* = 75)	(*N* = 33)	(*N* = 61)	(*N* = 31)	(*N* = 200)	
Age (Years)	65.6 ± 4.91	64.8 ± 4.60	66.7 ± 5.87	68.4 ± 6.70 ±	66.2 ± 5.55	0.111
Sex (Male)	50 (66.7%)	21 (63.6%)	48 (78.7%)	23 (74.2%)	142 (71.0%)	0.327
**Education Level**						
Illiterate	7 (9.3%)	2 (6.1%)	6 (9.8%)	4 (12.9%)	19 (9.5%)	0.24
Primary	9 (12.0%)	3 (9.1%)	8 (13.1%)	9 (29.0%)	29 (14.5%)	
Elementary	14 (18.7%)	6 (18.2%)	9 (14.8%)	3 (9.7%)	32 (16.0%)	
High school	15 (20.0%)	10 (30.3%)	6 (9.8%)	8 (25.8%)	39 (19.5%)	
University	25 (33.3%)	9 (27.3%)	24 (39.3%)	5 (16.1%)	63 (31.5%)	
Postgraduate	5 (6.7%)	3 (9.1%)	8 (13.1%)	2 (6.5%)	18 (9.0%)	
Diabetes	36 (48.0%)	10 (30.3%)	34 (55.7%)	16 (51.6%)	96 (48.0%)	0.128
Hypertension	46 (61.3%)	16 (48.5%)	38 (62.3%)	20 (64.5%)	120 (60.0%)	0.54
CAD	16 (21.3%)	3 (9.1%)	10 (16.4%)	5 (16.1%)	34 (17.0%)	0.501
Pain	37 (49.3%)	14 (42.4%)	25 (41.0%)	16 (51.6%)	92 (46.0%)	0.694
Stroke	1 (1.3%)	1 (3.0%)	5 (8.2%)	6 (19.4%)	13 (6.5%)	0.006
Depression	1 (1.3%)	1 (3.0%)	2 (3.3%)	0 (0%)	4 (2.0%)	0.688
Anxiety	2 (2.7%)	1 (3.0%)	2 (3.3%)	1 (3.2%)	6 (3.0%)	1
Nighttime Sleep (Hours)	6.00 ± 1.85	6.03 ± 1.57	5.91 ± 1.57	6.19 ± 2.46	6.01 ± 1.82	0.944
Nap time (Minutes)	0	25.7 ± 7.42	67.4 ± 14.9	157 ± 52.9	49.1 ± 58.4	N/A
Total SLUMS score	24.4 ± 4.06	25.4 ± 3.20	24.5 ± 4.38	21.0 ± 6.85	24.1 ± 4.74	0.0159

Cognitive function between non-, short, moderate and extended nappers was compared. Using a multiple linear regression model, the extended nappers had a significant lower SLUMS score compared to non-nappers [−2.26 units; 95% CI (−3.76, −0.76), *p* = 0.004] after controlling for the covariates (age, sex, education level, sleep hours, DM, HTN, pain, risk of insomnia and risk of sleep apnea). This indicates worse cognitive function among extended nappers.

Table 2 shows the multiple linear regression analysis. We found significant correlation between total SLUMS score which indicate cognitive function and age, sex, education level, and extended napping time.

**TABLE 2:** Multiple linear Regression dependent variable total SLUMS score.

Constant	26.18** (19.49, 32.86)
Age	−0.13** (−0.22, −0.03)
Sex (Male)	1.62** (0.52, 2.72)
Education: Primary	4.32** (2.26, 6.39)
Education: Elementary	6.37** (4.33, 8.41)
Education: High school	7.70** (5.64, 9.76)
Education: University	8.27** (6.35, 10.20)
Education: Postgraduate	9.60** (7.27, 11.93)
Sleep hour	−0.22 (−0.49, 0.04)
DM: Yes	−0.66 (−1.72, 0.40)
Hypertension: Yes	0.44 (−0.63, 1.50)
Coronary Artery Disease: Yes	0.94 (−0.42, 2.30)
Short nappers	0.70 (−0.73, 2.13)
Moderate nappers	−0.02 (−1.21, 1.17)
Extended nappers	−2.16** (−3.66, −0.66)
Observations	200
*R* ^2^	0.51
Adjusted *R*^2^	0.48
Residual Std. Error	3.43 (df = 185)
F Statistic	13.87** (df = 14; 18)

**p < 0.01.

## Discussion

The primary finding of this study was that the extended napping among Saudi older adults was associated with deterioration in cognitive function using SLUMS score.

In the local society, afternoon nap is a common practice (Bahammam et al., 2005; Al-Abri et al., 2020). Multiple studies have reported that most of the elderly nappers (> 60%) usually take moderate (30–60 min) to long (> 60 min) nap duration (Komada et al., 2012; Zhou et al., 2016; Faraut et al., 2017). However, data are still inconsistent on whether older adults take longer naps compared to other age groups. For instance, a Japanese study of 7,664 adults (20–99 years-old) revealed that longer nap durations were less frequently reported among those who are older in age (Furihata et al., 2016). On the other hand, a population-based study conducted on 12,277 participants in China showed that old adults are more likely to take naps with longer duration (Yin et al., 2018).

Our findings are consistent with several studies that showed extended napping was associated with poor cognitive performance. The association between nap duration and cognitive functions has been a focus of attention in multiple studies (Zhang et al., 2020). For instance, a study of 2,751 elderly participants showed that men who napped for longer duration were more likely to have cognitive decline over time (Leng et al., 2019). In addition, according to a study published in the Journal of the American Geriatrics Society by Li et al. (2017), it was reported that while a 30- to 90-min nap in older adults appears to have cognitive benefits, anything longer than an hour and a half may diminish these benefits. In a nationally representative American study, researchers also found that older adults who take long naps (> 60 min) had worse cognitive performance, while moderate-duration nappers (31–60 min) performed better on delayed word recall task (DWR) than short-nappers (< 30 min) (Owusu et al., 2019). Another study conducted on elderly women in United States reported that napping for 2 h or more a day was associated with increased risk of cognitive impairment (Blackwell et al., 2006). Furthermore, a study on 3,037 Chinese older adults (> 60 years-old) showed that self-reported naps of moderate duration was associated with better cognition, while no-napping or extended napping (> 90 min) was correlated with cognitive decline over 2 years (Li et al., 2018). These results suggest a U-shaped relationship between nap length and cognitive performance in older adults. A large Chinese study of approximately 11,000 elderly participants observed a 34% increase in odds of cognitive impairment among short nappers and towfolds increase in risk of cognitive deterioration among nappers of long duration (Lin et al., 2018). These cognitive benefits of moderate napping seems to benefit both habitual and non-habitual nappers (Leong et al., 2021).

The exact reason for this cognitive decline in long nappers is still under investigation. However, there are multiple mechanisms that may enlighten this association between napping and cognitive decline. One explanation is that extended nappers try to compensate their low-quality sleep by spending more time napping, and sleep disturbance has been reciprocally linked with deposition of beta amyloid (biomarker of Alzheimer disease) and vice versa (Ju et al., 2014). Another explanation is that the sleep characteristics in old adults is disturbed dramatically. For example, the amount of slow wave sleep is diminished leading to poor sleep quality and consequently frequent napping (Blackwell et al., 2006). It might be possible that frequent napping in older adults can prerequisites clinical diagnosis of Alzheimer disease (Owusu et al., 2019).

Extending afternoon napping can lead to delayed shift in nocturnal sleep, and therefore impair circadian rhythm and alter homeostasis. Indeed, previous studies have shown that long afternoon napping was associated with increased risk of cardio metabolic disorders (Fang et al., 2013). Furthermore, confounding factor such as diabetes mellitus, cardiac and/or cerebrovascular diseases can be factors that contribute to cognitive decline, yet these factors have been controlled for in our study (Kalaria, 2012).

There were some limitations in this study. First, the main focus of this study was on the duration of the nap. Therefore, the frequency and timing of the nap were not explored. Although majority of previous studies mainly focused in the duration, the frequency and timing of the nap are also important factors. Second, the assessment of nap duration was based on subjective self-report data and therefore, these data might be recall biased.

Despite these limitations, this study has several strengths. To our knowledge, this is the first study conducted in the region that assessed the relationship between nap duration and cognition among old adults. The SLUMs score used in this study is a validated clinical tool that can diagnose older adults with dementia. Also, the data was collected through a face-to-face questionnaire limiting the rate of dropout to only 3%.

Studies on the prevalence of cognitive impairment in Saudi Arabia are scarce. A community-based study of 171 Saudi old adults in Riyadh revealed an estimated prevalence of 38.6% of old adults having mild cognitive impairment (MCI) and 6.4% of them having dementia, as determined by the Montreal Cognitive Assessment (Alkhunizan et al., 2018). Without larger nationally representative studies, it is difficult to generalize these results for the Saudi population. Hence, further research is needed to fill this gap.

## Conclusion

Our findings showed that the extended napping was associated with deterioration in cognitive functions among Saudi old adults. Furthermore, most of our participants were non-nappers followed by moderate nappers. We believe that this study will provide new insights of older adults napping behavior and its relationship with cognitive decline among Saudi older adults. The study also sheds a light on the importance of monitoring the behavior of old adults; their extended napping may be associated with a more severe form of dementia.

## Data Availability Statement

The raw data supporting the conclusions of this article will be made available by the authors, without undue reservation.

## Ethics Statement

The studies involving human participants were reviewed and approved by the Institutional Review Board of Imam Abdulrahman bin Faisal University (IRB Number -2021-01-129). The patients/participants provided their written informed consent to participate in this study.

## Author Contributions

All authors listed have made a substantial, direct, and intellectual contribution to the work, and approved it for publication.

## Conflict of Interest

The authors declare that the research was conducted in the absence of any commercial or financial relationships that could be construed as a potential conflict of interest.

## Publisher’s Note

All claims expressed in this article are solely those of the authors and do not necessarily represent those of their affiliated organizations, or those of the publisher, the editors and the reviewers. Any product that may be evaluated in this article, or claim that may be made by its manufacturer, is not guaranteed or endorsed by the publisher.
